# Opinion: Bridging gaps and doubts in glioblastoma cell-of-origin

**DOI:** 10.3389/fonc.2022.1002933

**Published:** 2022-10-21

**Authors:** Nicolina Jovanovich, Ahmed Habib, Jeffery Head, Austin Anthony, Lincoln Edwards, Pascal O. Zinn

**Affiliations:** ^1^ Hillman Cancer Center, University of Pittsburgh Medical Center, Pittsburgh, PA, United States; ^2^ Department of Neurosurgery, University of Pittsburgh Medical Center, Pittsburgh, PA, United States

**Keywords:** SVZ, glioma, origin, GBM, opinion

## Introduction

Glioblastoma (GBM) remains one of the deadliest forms of brain cancer to date, with current patient survival stagnating at 12-15 months with treatment (1, 2). Extensive cellular heterogeneity within these neoplasms results from malignant cells in multiple cellular states that dynamically react to their microenvironment (3–6). The described heterogeneity within and between these tumors is believed to dictate the high treatment resistance and fatality rates of these central nervous system (CNS) malignancies (7–9). Drug screening has revealed significant differences in the response of genetically heterogeneous GSC cultures to cancer therapeutics, marking a significant barrier for establishing a standard of care that universally improves clinical outcomes (10, 11).

Intra-tumoral heterogeneity of GBM has been attributed to clonal evolution—in which accumulation of mutations in the cell of origin leads to the development of multiple, cancerous clones with differing therapeutic sensitivities and ability to survive—as well dynamic interactions between GSCs and their tumor microenvironment that can lead to niches with adaptive changes in their epigenetic landscapes (4, 12–15). While these processes can also contribute to inter-tumoral heterogeneity, the ability of different cells of origin to contribute to this heterogeneity has also been discussed (16–18). Various models of GBM have shown there to be multiple distinct neural/glial populations with oncogenic potential. Furthermore, genetic profiling of these distinct models has shown that transcriptionally active oncogenic programs, and thus, tumor drug sensitivity, is cell of origin dependent (16, 18, 19). Being able to understand and identify these cells of origin, as well as their effects on the clinical course of GBM patients, could be crucial in developing more effective therapeutics for this patient population. This article aims to review evidence supporting various cell types as cells of origin for GBM, as well as the niche that likely gives rise to these GSCs: the subventricular zone(SVZ).

## Evaluation of the candidacy of different stem cell populations as glioblastoma cell of origin

### Neural stem cells

Historically, it has been suggested that stem cells are the most likely candidate for a GBM cell of origin given their proliferative capacity (20–23). A recent study, in which *Nf1*, *Trp53*, and *PTEN* mutations were targeted to cells at various stages in the neural stem cell (NSC) lineage, was done to test the tumorigenic capacity of stem cells versus more differentiated cells (24). Targeting mutations of these genes to adult neurons (Cam2ka) and immature neurons (Neurod1) did not show any evidence of oncogenic potential, with immunohistochemistry staining (IHC) showing no tumorigenesis or histological abnormalities. When these mutations were induced in late-stage neural progenitors(iDix), abnormal cell proliferation was noted in the subventricular zone and rostral migratory stream, although no intracranial tumors resulted. This was believed to signify a pre-tumorigenic state. Targeting of these mutations to neural stem cells and oligodendrocyte-precursor cells (Syn-1) alone showed oncogenic potential, with intracranial tumors being observed and histological analysis showing a staining pattern consistent with GBM: positive for GFAP, Olig2, Sox2, PDGFR-alpha. Visualization of the tumors revealed anatomic similarities to Type II GBM (24). These data suggest that stem cells are most susceptible to oncogenic mutations, making them more likely candidates for a GBM cell of origin than more differentiated cells resistant to tumorigenesis. Various other models have confirmed the ability of mutated NSCs to initiate gliomagenesis (16, 25–27).

The presence of an NSC-like population driving gliomagenesis in GBM has been confirmed *via* time-series analyses of GBM models. Integration of whole-exome sequencing and bulk- and single-cell RNA-seq have identified a population of cycling NSCs in these models that persist throughout the entirety of gliomagenesis, unlike non-cycling and adult NSCs that drop in percentage over time. These cycling NSCs were enriched in mesenchymal-like and OPC-like markers often seen in end-stage GBM, validating their potential role in human gliomagenesis (28).

### Oligodendrocyte progenitor cells

The presence of an oligodendrocyte-like signature in end-stage GBM has led to the investigation of OPCs as a possible cell of origin (16, 28, 29). In 2011, Liu et al. initiated p53 and Nf1 concurrently into NSCs. The formation of GFP+ tumors occurred around 5 months of age. In order to determine the cell of origin for these tumors, a ratio of green (mutant) to red (WT) cells was determined for each cell type. OPCs were found to have the largest growth advantage (G/R > 130), corroborating the enriched expression of OPC markers (Olig2+, CD9, NG2, and PDGFRalpha) in this GBM tumors (30). Subsequent analyses have confirmed the presence of a population of OPC-intermediates in GBM patients that are reprogrammed to a stem cell-like state, leading to rapid proliferation and increased oncogenic susceptibility (31).

Given that adult, OPCs proliferate much less than their neonatal counterparts, the oncogenic potential of these more quiescent cells has been studied to validate this much more robust population as a possible origin of GBM. Induction of concurrent p53 and NF1 mutations into adult OPCs by Galvao et al. showed a consistent formation of gliomas. Tracking of these mutated adult-OPCs revealed a multi-step reactivation process, in which an increase in proliferative rate was followed by a period of dormancy that led to eventual gliomagenesis. Hematoxylin and eosin (H&E) staining confirmed the high cellularity and invasiveness of these tumors, which are features typical of GBMs, validating the oncogenic potential of these OPCs (32).

Recent miRNA surveillance of GBM tumors also revealed that oligodendrocyte lineage cells (Olig2+, NG2+, O4+) and macrophages (Iba1+, CD163+) were increased at the border of these tumors, with three of the top miRNAs (miR-219-5p, miR-219-2-3p, and miR-338-3p) in this region relating to oligodendrocyte differentiation (33). Co-culturing TMZ-sensitive GBM cells with conditioned medium (CM) from OPCs and macrophages increased chemotherapy resistance in these cells, resulting in a cell viability increase from 70% to 85% (p<0.05) (33). Such data suggest that OPCs may play an imperative role in not only initiating tumorigenesis but also maintaining the proliferative state of GSCs in GBM patients.

### Multiple cells of origin

While a stem cell population is widely accepted as the likely cell of origin, the inter- and intra-heterogeneity seen in GBM provides the possibility of there being more than one cell of origin. In 2011, Lai et al. compared the radiographic, anatomic, and genomic/transcriptomic characteristics of human isocitrate dehydrogenase mutant (IDH^R132MUT^) GBMs to isocitrate dehydrogenase wild type (IDH^WT^) GBMs (18). Analysis revealed that IDH^R132MUT^ GBMs were found to have a significant predominance in the frontal lobe (p<0.0001) when compared to their IDH^WT^ counterpart, especially surrounding the rostral extension of the lateral ventricle. Transcriptomic data confirmed differences between the two groups, as well, with IDH^R132MUT^ GBMs predominantly expressing a pro-neural subtype and IDH^WT^ GBMs—although they were found to have a variety of signatures – expressing a predominantly mesenchymal subtype. This data, in combination with distinct age-frequency patterns between the two types of GBMs, suggests that IDH^R132MUT^ GBMs and IDH^WT^GBMs are spatially and temporally restricted, and thus, most likely arise from different cells of origin (18). A recent study using multi-omics to characterize the heterogeneity of glioma cells was similarly able to distinguish five-spatially distinct lineage states (glial-related, radial glia/inflammatory, NPC, OPC, and “reactive-hypoxia”) within and between GBM patients (4). Analysis of these niches showed that each spatially distinct population played distinct pro-tumorigenic roles within the GBM tumor, with the “reactive-hypoxia” cells supporting resilience through enhancement of genomic instabilities and the “reactive-immune” cells increasing cell-to-cell communication with the immune compartment. The presence of multiple stem cell-like populations (NPC, OPC, radial glia) within and between tumors highlights the challenge of delineating a single cell of origin for GBM. While some transcriptional programs (reactive hypoxia and immune) seemed to arise from interactions of the tumor with its microenvironment, it is unclear whether the different stem cell lineages within these tumors arose from their distinct lineages or by proxy of epigenetic dysregulation in one cell of origin (4).

**Figure 1:**
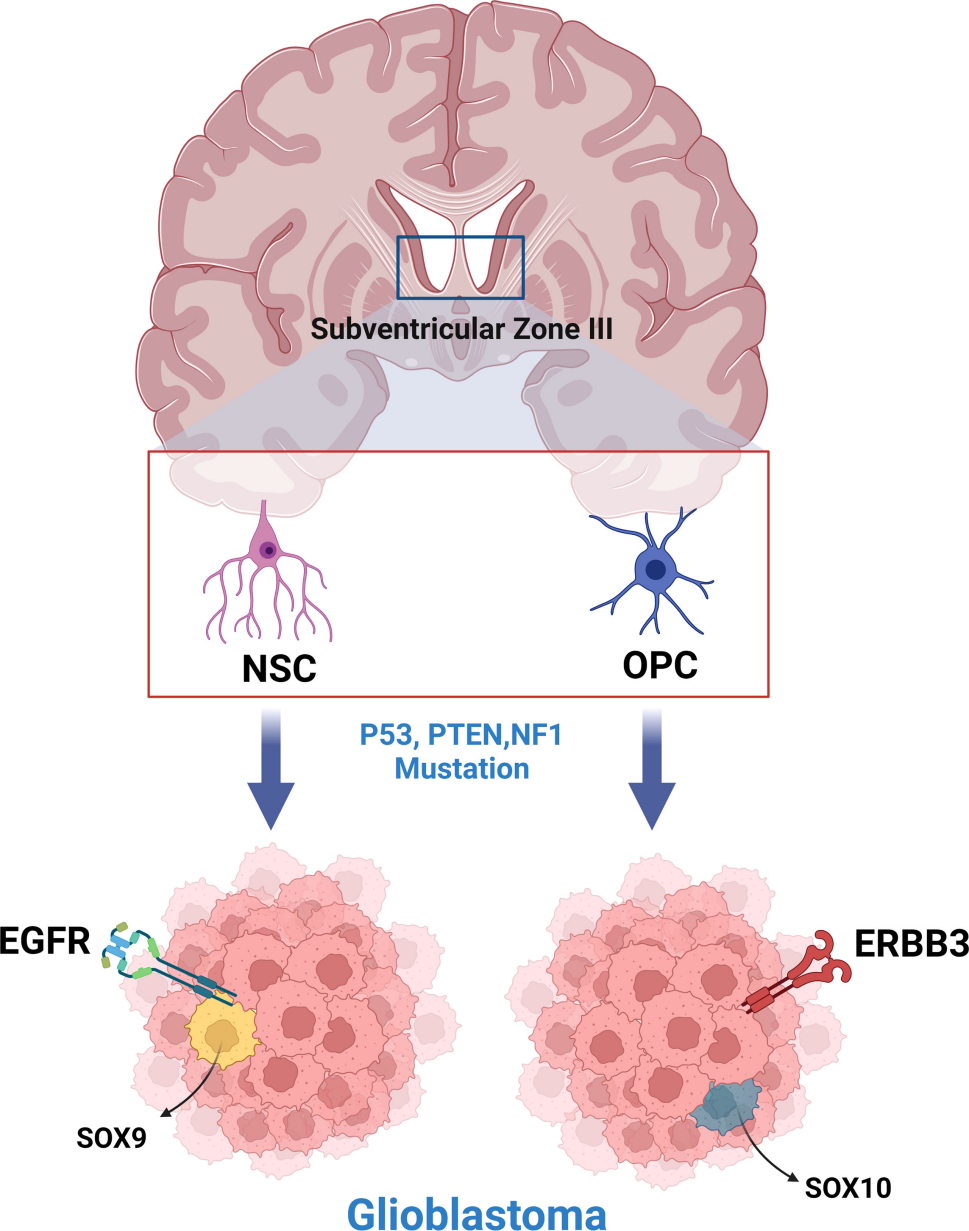
Neural stem cells and oligodendrocyte progenitor cells of the subventricular zone produce phenotypically and molecularly distinct glioblastoma tumors when engineered to express common glioblastoma driver mutations.

Subsequent models of GBM created using various tumor-initiating cells (NSCs, OPCs, astrocytes) have further supported the hypothesis of multiple cells of origin for GBM (19, 25, 30, 32). In a study by Wang et. al., genetically engineered mouse models (GEMMs) were created by inducing common GBM-driver mutations in NSCs and OPCs (16). Transcriptome analysis revealed that GBMs arising from each cell lineage had distinct phenotypic and molecular differences, with NSC initiated tumors expressing EGFR/SOX9+ (Type I) and OPC initiated tumors expressing ERBB3/Sox10+ (Type II) (**Figure 1**). These gene expression signatures were analyzed in a human GBM database, with 107 Type I candidates and 68 Type II candidates being found (16). A similar study, in which astrocytes and neural stem/progenitor cells (NSPCs) were targeted with common GBM driver mutations, showed that tumors between the two models with different cells of origin had distinct latency to symptom timelines and protein expression (19). This indicates that latency and transcriptomic effects of driver mutations are likely cell of origin dependent and may explain the prevalence of GBM inter-tumor heterogeneity, both in terms of phenotype and clinical course.

## Subventricular zone as location of cell of origin niche

The subventricular zone (SVZ), which lies along the lateral ventricle, consists of multiple cell populations (astrocytic lineage [type B1 and B2], neuroblasts [type A], and transit-amplifying cells [type C]) that represent the largest reservoir of NSCs in the human brain (34, 35). Astrocytic-like NSCs give rise to transit-amplifying cells (TACs), which in turn produce neuroblasts that migrate throughout the SVZ and along the rostral migratory stream (RMS) to the olfactory bulb where they can differentiate into immature neurons (36). Many molecular characteristics are shared between glioblastoma stem cells (GSCs) and NSCs, making the SVZ the main suspect of GBM tumorigenesis and recurrence (7, 37).

## Genetic comparison of subventricular zone neural stem cells to glioma stem cells

Suspicion of the SVZ in GBM gliomagenesis has led to comparative studies between GSCs and NSCs residing in the SVZ. In a study done by Lee et. al., deep-sequencing of matched tissues (non-tumor SVZ, glioblastoma tumor, and cortex) from IDH^WT^ GBM patients showed that 56.3% of these patient’s SVZs expressed low levels of driver mutations observed in their tumors (38). A paired model where common GBM-driver mutations were induced in cortical cells showed no spread of these cells to the SVZ, confirming that the observed SVZ-cells with low-level driver mutations were the initiating population and not a migratory niche (38). Further analyses using immunofluorescence (IF) and IHC staining have confirmed the presence of GSC-like cells in the SVZ of GBM patients. These GSC-like cell niches stain positive for a combination of CD133; Sox2; CD9+, as well as GBM-related chemokines and receptors: stromal-derived factor-1alpha (SDF-1alpha), C-X-C receptor type 4 (CXC4), osteopontin, and CD44 (39). Moreover, common SVZ-related markers such as glial fibrillary acidic protein (GFAP) and vimentin have also been found to be highly expressed in GSCs in other models (37).

Subsequent transcriptome analyses of GSCs have identified a cell population in GBM tumors similar to outer radial glia (oRG): NSCs that are abundant in the SVZ during human cortical development (6, 40, 41). Live time-lapse imaging showed that these oRG-like GSCs underwent mitotic somal translocations typical of neonatal oRGs. Progeny of these GSCs were seen to undergo cell divisions of their own, demonstrating their ability to amplify a cell population and their potential to initiate tumorigenesis (6).

## Models of glioblastoma implicating subventricular zone neural stem cells in glioblastoma gliomagenesis

The initiation of tumors *via* genetic modifications of NSCs in the SVZ has been well studied. In one instance, p53 mutations were induced in mice *via* a Cre-flox system. Almost all of the p53^flox/flox^ and p53^flox/KO^ (85% and 84%, respectively) mice developed neurological symptoms (tremor, ataxia, etc.) that indicated high grade glioma (HGG) formation. Forty percent of these tumors showed characteristics common to GBM, such as necrosis and microvascular proliferation, confirming a GBM phenotype. Stained tumor cells exhibited high levels of Beta-galactosidase, an enzyme commonly expressed by SVZ NSCs and OPCs. To determine if these SVZ cells were the cell of origin, Wang et al. looked at which cell niches primarily accumulated the p53^delta5-6^ protein. Eighty percent of p53^delta5-6^-positive cells were found to be within the adult SVZ niche and seventy-seven percent of these cells were found to be actively dividing. Although most of these cells were labeled as SVZ-B stem cells, they were shown to be able to differentiate into Olig2+ SVZ-C progenitor cells, confirming their oncogenic potential (42). Other similar models inducing common-GBM driver mutations in SVZ NSCs have confirmed the oncogenic potential of this niche (38, 43, 44). In one of these studies, serial sections showed that over time, genetically engineered NSCs were able to migrate from the SVZ to various regions of the brain, such as the olfactory bulb and dorsolateral caudal cortex. Here, they proliferated and developed into high-grade malignant gliomas (38).

SVZ NSCs have been implicated not only in the initiation of GBM but also in its continued maintenance and recurrence. In a murine model of GBM, SVZ NSCs were shown to re-initiate GBM tumor growth after cell proliferation in the primary tumor was arrested using temozolomide (TMZ) (45). Ablation of these SVZ-niche cells in 8-week-old mutant mice resulted in significantly increased survival and a decrease in overall tumor progression (45). Similarly, in a clinical trial where GBM patients had the SVZ ipsilateral to their tumor treated with radiation, patients who received the treatment had significantly longer progression-free survival (PFS; 12.6 *vs.* 9.9 months, p=0.042) and longer overall survival (OS;25.8 *vs.* 19.2 months, p=0.173) when compared to patients who received no radiation to their SVZ (46). These data, as well as data from other studies, suggest that GSCs from the SVZ-niche may be able to re-populate primary tumors after treatment and lead to GBM recurrence (47).

Contact of a patient’s primary GBM tumor with the SVZ has also been indicated as a prognostic factor of patient survival. In a study by Jafri et. al., GBM tumors were classified based off of their contact with both the cortex and the SVZ (type I), just the SVZ (type II), just the cortex (type III), and neither the cortex nor the SVZ (type IV). Patients with tumors involving the SVZ (type I & II) had a poorer two-year OS (23% *vs.* 48%; p=0.0002) and more rapid time to progression (p=0.003) than patients with tumors not involving the SVZ (type III & IV) (48). Multiple studies have since linked GBM tumor contact with the SVZ to shortened time to recurrence and poorer prognosis, possibly due to the findings that SVZ contact with GSCs initiates enrichment of pro-oncogenic programs, such as the epithelial to mesenchymal transition (EMT), NF-kBeta, angiogenesis, and hypoxia gene sets (49–52).

## Discussion and final remarks

A definitive cell of origin for GBM has still not been identified. The presence of a large, proliferative niche of NSCs in the SVZ of the adult brain, as well as the accumulating evidence of a stable, cycling NSC population in GBM models, continues to support NSCs as the most likely cell or origin (7, 24, 28, 34, 38). However, multi-omics analyses of genetically engineered models and patient samples suggest that differences in GBM-subtypes may be correlated to transcriptionally active oncogenic pathways that are cell of origin dependent, suggesting that multiple cells of origin may be responsible for the inter-tumoral heterogeneity seen in GBM (16, 19, 32). Better profiling of these cell of origin dependent effects could help with understanding the therapeutic vulnerabilities of genetically distinct tumors between patients, just as other molecular profiling markers have (53, 54).

Recent evidence showing that some GBM patients express low levels of their cancer’s driver mutations in their SVZ, as well as the discovery of a population of GSCs that behave similarly to oRG, supports the theory that GBM may develop from NSCs in the SVZ (6, 38). Subsequent models have confirmed the oncogenic potential of this stem cell niche, as well as its possible involvement in the maintenance and recurrence of GBM tumors after treatment (42, 45). Reports on the clinical efficacy of treating GBM patients’ SVZs with radiation therapy are conflicting, with some studies showing clear clinical benefits and some showing none (47, 55, 56). The need to initiate further clinical trials studying the effect of RT of the SVZ on GBM patient outcomes is of utmost importance.

Various studies have also elucidated the importance of SVZ to GBM cell-cell interactions in driving gliomagenesis and contributing to poorer outcomes (48, 50, 51, 57, 58). CXCL12/CXCR4 signaling seems to be of particular importance in initiating more extensive migration of GSCs, thus, promoting further malignancy (57). Elucidation of these complex, 3D interactions *via* cerebral organoids would provide a means for identifying molecular patterns and therapeutic vulnerabilities of GSCs in a model that better replicates the complexity of the human brain (59–61).

The cell of origin, as well as the SVZ, play an important role in GBM initiation, maintenance, and recurrence that, to this day, is still not entirely understood. It remains of great interest across the field of neuro-oncology to focus a significant portion of all future GBM research on uncovering the molecular consequences of the cell of origin differences and SVZ to tumor interactions and how these consequences subsequently affect therapeutic susceptibility and treatment courses of GBM tumors. The ability to sub-type GBMs based off the aforementioned characteristics could greatly improve the accuracy of GBM diagnosis and treatment, and subsequently, improve patient outcomes.

## Author contributions

Conception and design: PZ. Interpretation of data: PZ, NJ, and AH. Drafted the manuscript: NJ and AH. Approved the final version to be published: PZ. Agree to be accountable for all aspects of the work in ensuring that questions related to the accuracy or integrity of any part of the work are appropriately investigated and resolved: PZ. All authors contributed to the article and approved the submitted version.

## Funding

UPMC University of Pittsburgh medical center startup funds.

## Conflict of interest

The authors declare that the research was conducted in the absence of any commercial or financial relationships that could be construed as a potential conflict of interest.

## Publisher’s note

All claims expressed in this article are solely those of the authors and do not necessarily represent those of their affiliated organizations, or those of the publisher, the editors and the reviewers. Any product that may be evaluated in this article, or claim that may be made by its manufacturer, is not guaranteed or endorsed by the publisher.

## References

[1] Ostrom QT Cioffi G Waite K Kruchko C Barnholtz-Sloan JS. CBTRUS statistical report: Primary brain and other central nervous system tumors diagnosed in the united states in 2014-2018. Neuro Oncol (2021) 23(12 Suppl 2):iii1–105. doi: 10.1093/neuonc/noab200 34608945 PMC8491279

[2] Stylli SS. Novel treatment strategies for glioblastoma. Cancers (Basel) (2020) 12(10):2883. doi: 10.3390/cancers12102883 PMC7599818 33049911

[3] Neftel C Laffy J Filbin MG Hara T Shore ME Rahme GJ. An integrative model of cellular states, plasticity, and genetics for glioblastoma. Cell (2019) 178(4):835–49.e21. doi: 10.1016/j.cell.2019.06.024 31327527 PMC6703186

[4] Ravi VM Will P Kueckelhaus J Sun N Joseph K Salié H. Spatially resolved multi-omics deciphers bidirectional tumor-host interdependence in glioblastoma. Cancer Cell (2022) 40(6):639–55.e13. doi: 10.1016/j.ccell.2022.05.009 35700707

[5] Tirosh I Venteicher AS Hebert C Escalante LE Patel AP Yizhak K. Single-cell RNA-seq supports a developmental hierarchy in human oligodendroglioma. Nature (2016) 539(7628):309–13. doi: 10.1038/nature20123 PMC5465819 27806376

[6] Bhaduri A Di Lullo E Jung D Müller S Crouch EE Espinosa CS. Outer radial glia-like cancer stem cells contribute to heterogeneity of glioblastoma. Cell Stem Cell (2020) 26(1):48–63.e6. doi: 10.1016/j.stem.2019.11.015 31901251 PMC7029801

[7] Beiriger J Habib A Jovanovich N Kodavali CV Edwards L Amankulor N. The subventricular zone in glioblastoma: Genesis, maintenance, and modeling. Front Oncol (2022) 12:790976. doi: 10.3389/fonc.2022.790976 35359410 PMC8960165

[8] Becker AP Sells BE Haque SJ Chakravarti A. Tumor heterogeneity in glioblastomas: From light microscopy to molecular pathology. Cancers (Basel). (2021) 13(4):761. doi: 10.3390/cancers13040761 33673104 PMC7918815

[9] Qazi MA Vora P Venugopal C Sidhu SS Moffat J Swanton C. Intratumoral heterogeneity: Pathways to treatment resistance and relapse in human glioblastoma. Ann Oncol (2017) 28(7):1448–56. doi: 10.1093/annonc/mdx169 28407030

[10] Skaga E Kulesskiy E Fayzullin A Sandberg CJ Potdar S Kyttälä A. Intertumoral heterogeneity in patient-specific drug sensitivities in treatment-naïve glioblastoma. BMC Cancer. (2019) 19(1):628. doi: 10.1186/s12885-019-5861-4 31238897 PMC6593575

[11] Bao Z Wang Y Wang Q Fang S Shan X Wang J. Intratumor heterogeneity, microenvironment, and mechanisms of drug resistance in glioma recurrence and evolution. Front Med (2021) 15(4):551–61. doi: 10.1007/s11684-020-0760-2 33893983

[12] Parker NR Khong P Parkinson JF Howell VM Wheeler HR. Molecular heterogeneity in glioblastoma: Potential clinical implications. Front Oncol (2015) 5:55. doi: 10.3389/fonc.2015.00055 25785247 PMC4347445

[13] Greaves M Maley CC. Clonal evolution in cancer. Nature (2012) 481(7381):306–13. doi: 10.1038/nature10762 PMC3367003 22258609

[14] DeCordova S Shastri A Tsolaki AG Yasmin H Klein L Singh SK. Molecular heterogeneity and immunosuppressive microenvironment in glioblastoma. Front Immunol (2020) 11:1402. doi: 10.3389/fimmu.2020.01402 32765498 PMC7379131

[15] Sa JK Chang N Lee HW Cho HJ Ceccarelli M Cerulo L. Transcriptional regulatory networks of tumor-associated macrophages that drive malignancy in mesenchymal glioblastoma. Genome Biol (2020) 21(1):216. doi: 10.1186/s13059-020-02140-x 32847614 PMC7448990

[16] Wang Z Sun D Chen YJ Xie X Shi Y Tabar V. Cell lineage-based stratification for glioblastoma. Cancer Cell (2020) 38(3):366–79.e8. doi: 10.1016/j.ccell.2020.06.003 32649888 PMC7494533

[17] Yao M Li S Wu X Diao S Zhang G He H. Cellular origin of glioblastoma and its implication in precision therapy. Cell Mol Immunol (2018) 15(8):737–9. doi: 10.1038/cmi.2017.159 PMC6141605 29553137

[18] Lai A Kharbanda S Pope WB Tran A Solis OE Peale F. Evidence for sequenced molecular evolution of IDH1 mutant glioblastoma from a distinct cell of origin. J Clin Oncol (2011) 29(34):4482–90. doi: 10.1200/JCO.2010.33.8715 PMC3236649 22025148

[19] Ghazi SO Stark M Zhao Z Mobley BC Munden A Hover L. Cell of origin determines tumor phenotype in an oncogenic Ras/p53 knockout transgenic model of high-grade glioma. J Neuropathol Exp Neurol (2012) 71(8):729–40. doi: 10.1097/NEN.0b013e3182625c02 PMC3407564 22805776

[20] Kim HJ Park JW Lee JH. Genetic architectures and cell-of-Origin in glioblastoma. Front Oncol (2020) 10:615400. doi: 10.3389/fonc.2020.615400 33552990 PMC7859479

[21] Modrek AS Bayin NS Placantonakis DG. Brain stem cells as the cell of origin in glioma. World J Stem Cells (2014) 6(1):43–52. doi: 10.4252/wjsc.v6.i1.43 24567787 PMC3927013

[22] Modrek AS Prado J Bready D Dhaliwal J Golub D Placantonakis DG. Modeling glioma with human embryonic stem cell-derived neural lineages. Methods Mol Biol (2018) 1741:227–37. doi: 10.1007/978-1-4939-7659-1_19 29392705

[23] Zhang GL Wang CF Qian C Ji YX Wang YZ. Role and mechanism of neural stem cells of the subventricular zone in glioblastoma. World J Stem Cells (2021) 13(7):877–93. doi: 10.4252/wjsc.v13.i7.877 PMC8316865 34367482

[24] Alcantara Llaguno S Sun D Pedraza AM Vera E Wang Z Burns DK. Cell-of-origin susceptibility to glioblastoma formation declines with neural lineage restriction. Nat Neurosci (2019) 22(4):545–55. doi: 10.1038/s41593-018-0333-8 PMC6594191 30778149

[25] Zhu Y Guignard F Zhao D Liu L Burns DK Mason RP. Early inactivation of p53 tumor suppressor gene cooperating with NF1 loss induces malignant astrocytoma. Cancer Cell (2005) 8(2):119–30. doi: 10.1016/j.ccr.2005.07.004 PMC3024718 16098465

[26] Holland EC Celestino J Dai C Schaefer L Sawaya RE Fuller GN. Combined activation of ras and akt in neural progenitors induces glioblastoma formation in mice. Nat Genet (2000) 25(1):55–7. doi: 10.1038/75596 10802656

[27] Wang J Liu J Sun G Meng H Guan Y Yin Y. Glioblastoma extracellular vesicles induce the tumour-promoting transformation of neural stem cells. Cancer Lett (2019) 466:1–12. doi: 10.1016/j.canlet.2019.09.004 31521694

[28] Wang X Zhou R Xiong Y Zhou L Yan X Wang M. Sequential fate-switches in stem-like cells drive the tumorigenic trajectory from human neural stem cells to malignant glioma. Cell Res (2021) 31(6):684–702. doi: 10.1038/s41422-020-00451-z 33390587 PMC8169837

[29] Lindberg N Kastemar M Olofsson T Smits A Uhrbom L. Oligodendrocyte progenitor cells can act as cell of origin for experimental glioma. Oncogene (2009) 28(23):2266–75. doi: 10.1038/onc.2009.76 19421151

[30] Liu C Sage JC Miller MR Verhaak RG Hippenmeyer S Vogel H. Mosaic analysis with double markers reveals tumor cell of origin in glioma. Cell (2011) 146(2):209–21. doi: 10.1016/j.cell.2011.06.014 PMC3143261 21737130

[31] Weng Q Wang J He D Cheng Z Zhang F Verma R. Single-cell transcriptomics uncovers glial progenitor diversity and cell fate determinants during development and gliomagenesis. Cell Stem Cell (2019) 24(5):707–23.e8. doi: 10.1016/j.stem.2019.03.006 30982771 PMC6669001

[32] Galvao RP Kasina A McNeill RS Harbin JE Foreman O Verhaak RG. Transformation of quiescent adult oligodendrocyte precursor cells into malignant glioma through a multistep reactivation process. Proc Natl Acad Sci USA. (2014) 111(40):E4214–23. doi: 10.1073/pnas.1414389111 PMC4210043 25246577

[33] Hide T Komohara Y Miyasato Y Nakamura H Makino K Takeya M. Oligodendrocyte progenitor cells and Macrophages/Microglia produce glioma stem cell niches at the tumor border. EBioMedicine (2018) 30:94–104. doi: 10.1016/j.ebiom.2018.02.024 29559295 PMC5952226

[34] Lacar B Young SZ Platel JC Bordey A. Imaging and recording subventricular zone progenitor cells in live tissue of postnatal mice. Front Neurosci (2010) 4. doi: 10.3389/fnins.2010.00043 PMC2918349 20700392

[35] Doetsch F García-Verdugo JM Alvarez-Buylla A. Cellular composition and three-dimensional organization of the subventricular germinal zone in the adult mammalian brain. J Neurosci (1997) 17(13):5046–61. doi: 10.1523/JNEUROSCI.17-13-05046.1997 PMC6573289 9185542

[36] Curtis MA Kam M Nannmark U Anderson MF Axell MZ Wikkelso C. Human neuroblasts migrate to the olfactory bulb *via* a lateral ventricular extension. Science (2007) 315(5816):1243–9. doi: 10.1126/science.1136281 17303719

[37] Haskins WE Zablotsky BL Foret MR Ihrie RA Alvarez-Buylla A Eisenman RN. Molecular characteristics in MRI-classified group 1 glioblastoma multiforme. Front Oncol (2013) 3:182. doi: 10.3389/fonc.2013.00182 23875172 PMC3708153

[38] Lee JH Lee JE Kahng JY Kim SH Park JS Yoon SJ. Human glioblastoma arises from subventricular zone cells with low-level driver mutations. Nature (2018) 560(7717):243–7. doi: 10.1038/s41586-018-0389-3 30069053

[39] Hira VVV Molenaar RJ Breznik B Lah T Aronica E Van Noorden CJF. Immunohistochemical detection of neural stem cells and glioblastoma stem cells in the subventricular zone of glioblastoma patients. J Histochem Cytochem (2021) 69(5):349–64. doi: 10.1369/0022155421994679 PMC8091546 33596115

[40] Andrews MG Subramanian L Kriegstein AR. mTOR signaling regulates the morphology and migration of outer radial glia in developing human cortex. Elife (2020) 9. doi: 10.7554/eLife.58737 PMC7467727 32876565

[41] Hu Y Jiang Y Behnan J Ribeiro MM Kalantzi C Zhang MD. Neural network learning defines glioblastoma features to be of neural crest perivascular or radial glia lineages. Sci Adv (2022) 8(23):eabm6340. doi: 10.1126/sciadv.abm6340 35675414 PMC9177076

[42] Wang Y Yang J Zheng H Tomasek GJ Zhang P McKeever PE. Expression of mutant p53 proteins implicates a lineage relationship between neural stem cells and malignant astrocytic glioma in a murine model. Cancer Cell (2009) 15(6):514–26. doi: 10.1016/j.ccr.2009.04.001 PMC2721466 19477430

[43] Chen Z Herting CJ Ross JL Gabanic B Puigdelloses Vallcorba M Szulzewsky F. Genetic driver mutations introduced in identical cell-of-origin in murine glioblastoma reveal distinct immune landscapes but similar response to checkpoint blockade. Glia (2020) 68(10):2148–66. doi: 10.1002/glia.23883 PMC7512141 32639068

[44] Gil-Perotin S Marin-Husstege M Li J Soriano-Navarro M Zindy F Roussel MF. Loss of p53 induces changes in the behavior of subventricular zone cells: implication for the genesis of glial tumors. J Neurosci (2006) 26(4):1107–16. doi: 10.1523/JNEUROSCI.3970-05.2006 PMC6674560 16436596

[45] Chen J Li Y Yu TS McKay RM Burns DK Kernie SG. A restricted cell population propagates glioblastoma growth after chemotherapy. Nature (2012) 488(7412):522–6. doi: 10.1038/nature11287 PMC3427400 22854781

[46] Lee P Eppinga W Lagerwaard F Cloughesy T Slotman B Nghiemphu PL. Evaluation of high ipsilateral subventricular zone radiation therapy dose in glioblastoma: A pooled analysis. Int J Radiat Oncol Biol Phys (2013) 86(4):609–15. doi: 10.1016/j.ijrobp.2013.01.009 23462418

[47] Şuşman S Leucuţa DC Kacso G Florian ŞVerifytat. High dose vs low dose irradiation of the subventricular zone in patients with glioblastoma-a systematic review and meta-analysis. Cancer Manag Res (2019) 11:6741–53. doi: 10.2147/CMAR.S206033 PMC6645358 31410064

[48] Jafri NF Clarke JL Weinberg V Barani IJ Cha S. Relationship of glioblastoma multiforme to the subventricular zone is associated with survival. Neuro Oncol (2013) 15(1):91–6. doi: 10.1093/neuonc/nos268 PMC3534420 23095230

[49] Dalemans DJZ Berendsen S Draaisma K Robe PA Snijders TJ. Glioblastomas within the subventricular zone are region-specific enriched for mesenchymal transition markers: An intratumoral gene expression analysis. Cancers (Basel). (2021) 13(15):3764. doi: 10.3390/cancers13153764 34359668 PMC8345101

[50] Huang R Wang T Liao Z Wang Z Ye M Zhou D. A retrospective analysis of the risk factors affecting recurrence time in patients with recurrent glioblastoma. Ann Palliat Med (2021) 10(5):5391–9. doi: 10.21037/apm-21-823 34044551

[51] Kappadakunnel M Eskin A Dong J Nelson SF Mischel PS Liau LM. Stem cell associated gene expression in glioblastoma multiforme: Relationship to survival and the subventricular zone. J Neurooncol. (2010) 96(3):359–67. doi: 10.1007/s11060-009-9983-4 PMC2808508 19655089

[52] Chen L Chaichana KL Kleinberg L Ye X Quinones-Hinojosa A Redmond K. Glioblastoma recurrence patterns near neural stem cell regions. Radiother Oncol (2015) 116(2):294–300. doi: 10.1016/j.radonc.2015.07.032 26276527 PMC4857206

[53] Oh S Yeom J Cho HJ Kim JH Yoon SJ Kim H. Integrated pharmaco-proteogenomics defines two subgroups in isocitrate dehydrogenase wild-type glioblastoma with prognostic and therapeutic opportunities. Nat Commun (2020) 11(1):3288. doi: 10.1038/s41467-020-17139-y 32620753 PMC7335111

[54] Hu B Ruan Y Wei F Qin G Mo X Wang X. Identification of three glioblastoma subtypes and a six-gene prognostic risk index based on the expression of growth factors and cytokines. Am J Transl Res (2020) 12(8):4669–82. PMC7476164 32913540

[55] Hallaert G Pinson H Van den Broecke C Sweldens C Van Roost D Kalala JP. Survival impact of incidental subventricular zone irradiation in IDH-wildtype glioblastoma. Acta Oncol (2021) 60(5):613–9. doi: 10.1080/0284186X.2021.1893899 33689536

[56] Murchison SC Wiksyk B Gossman S Jensen B Sayers D Lesperance M. Subventricular zone radiation dose and outcome for glioblastoma treated between 2006 and 2012. Cureus (2018) 10(11):e3618. doi: 10.7759/cureus.3618 30697499 PMC6347443

[57] Goffart N Kroonen J Di Valentin E Dedobbeleer M Denne A Martinive P. Adult mouse subventricular zones stimulate glioblastoma stem cells specific invasion through CXCL12/CXCR4 signaling. Neuro Oncol (2015) 17(1):81–94. doi: 10.1093/neuonc/nou144 25085362 PMC4483049

[58] Lawlor K Marques-Torrejon MA Dharmalingham G El-Azhar Y Schneider MD Pollard SM. Glioblastoma stem cells induce quiescence in surrounding neural stem cells via notch signaling. Genes Dev (2020) 34(23-24):1599–604. doi: 10.1101/gad.336917.120 PMC7706704 33184225

[59] Logan S Arzua T Canfield SG Seminary ER Sison SL Ebert AD. Studying human neurological disorders using induced pluripotent stem cells: From 2D monolayer to 3D organoid and blood brain barrier models. Compr Physiol (2019) 9(2):565–611. doi: 10.1002/cphy.c180025 30873582 PMC6705133

[60] Lancaster MA Renner M Martin CA Wenzel D Bicknell LS Hurles ME. Cerebral organoids model human brain development and microcephaly. Nature (2013) 501(7467):373–9. doi: 10.1038/nature12517 PMC3817409 23995685

[61] Wörsdörfer P IT Asahina I Sumita Y Ergün S. Do not keep it simple: recent advances in the generation of complex organoids. J Neural Transm (Vienna). (2020) 127(11):1569–77. doi: 10.1007/s00702-020-02198-8 PMC7577912 32385575

